# Endothelial Cell Culture Under Perfusion On A Polyester-Toner Microfluidic Device

**DOI:** 10.1038/s41598-017-11043-0

**Published:** 2017-09-05

**Authors:** Ana Carolina Urbaczek, Paulo Augusto Gomes Carneiro Leão, Fayene Zeferino Ribeiro de Souza, Ana Afonso, Juliana Vieira Alberice, Luciana Teresa Dias Cappelini, Iracilda Zeppone Carlos, Emanuel Carrilho

**Affiliations:** 1 Instituto de Química de São Carlos, IQSC, Universidade de São Paulo, USP, São Carlos, SP Brazil; 2 GHTM – Global Health and Tropical Medicine, Instituto de Higiene e Medicina Tropical, IHMT, Universidade Nova de Lisboa, UNL, Lisboa, Portugal; 3 Laboratório de Parasitologia, Departamento de Morfologia e Patologia, Universidade Federal de São Carlos, UFSCar, São Carlos, SP Brazil; 4 Escola Paulista de Medicina, Universidade Federal de São Paulo, Unifesp, São Paulo, SP Brazil; 5 Faculdade de Ciências Farmacêuticas, FCFar, Universidade Estadual Paulista, UNESP, Araraquara, SP Brazil; 6 Instituto Nacional de Ciência e Tecnologia de Bioanalítica, INCTBio, Campinas, SP Brazil

## Abstract

This study presents an inexpensive and easy way to produce a microfluidic device that mimics a blood vessel, serving as a start point for cell culture under perfusion, cardiovascular research, and toxicological studies. Endpoint assays (*i.e*., MTT reduction and NO assays) were used and revealed that the components making up the microchip, which is made of polyester and toner (PT), did not induce cell death or nitric oxide (NO) production. Applying oxygen plasma and fibronectin improved the adhesion and proliferation endothelial cell along the microchannel. As expected, these treatments showed an increase in vascular endothelial growth factor (VEGF-A) concentration profiles, which is correlated with adherence and cell proliferation, thus promoting endothelialization of the device for neovascularization. Regardless the simplicity of the device, our “vein-on-a-chip” mimetic has a potential to serve as a powerful tool for those that demand a rapid microfabrication method in cell biology or organ-on-a-chip research.

## Introduction

In multicellular organisms, tissues are formed by different types of cells that are organized three-dimensionally (3D) into cooperative assemblies. The different varieties of tissues, in turn, combine to form larger functional units called organs. In organ microenvironment, cells undergo specific-organ dynamic variation, such as chemical gradients, and mechanical forces (*i.e*., compression and fluid tension). Such forces are crucial for growth, survival, response, and functionality of cells, tissues, and organs. Thus, to understand human pathology, investigation of how cells and tissues perform all together their functions is of extreme importance^1, 2^.

Cell cultures are ubiquitous in cell biology, biochemistry, drug discovery and development, pharmacokinetic studies, and tissue engineering. Fundamental investigations of human biology and the development of therapeutic drugs, commonly rely on two-dimensional (2D) monolayer cell culture systems or animal models. However, 2D cell culture systems do not accurately recapitulate the structure, function, physiology of living tissues, as well as highly complex and dynamic 3D environments *in vivo*. For this reason, many studies involving physiology and pathology demand the use of animal models that have various problems, such as: high costs, ethical questions, and, in many cases, these models are not applicable to predict and reproduce human responses. Thus, animal models and *in vitro* models are ineffective models in predicting *in vivo* responses due to inter-species difference and/or lack of physiologically relevant 3D tissue environment^1–6^.

For this reason, limitations of cell culture usage and of existing animal models are the stimulus for the development of new *in vitro* alternative models that could better mimic the complex structural functionality of the organs. Thus, considerable advances are being made in this field as a result of microsystems engineering applications in cell cultures. Microfabrication techniques enable the development of microchips that allow an accurate control of the cell, position, function, and respective tissue organization in highly structured cultures^4, 7–9^. Moreover, when microfluidic technology is associated with microchips, this allows a more dynamic control of fluid flow and pressure, enabling the creation of a microenvironment surrounding the cells that originates various chemical gradients and dynamic mechanical signals, which are capable of inducing cell responses that closely mimics a physiological-like microenvironment^10, 11^.

Organ-on-a-chip can mimic *in vivo* like environment and subsequently *in vivo* like responses, generating a realistic model of human organs of interest, which can potentially provide human physiology-relevant models. The development of an organ-on-a-chip came from the merge of both micro engineering methods, such as, microfluidics technology and cellular biology^2^. Organs-on-a-chip reconstitute structural arrangements of the tissues and resembles the functional complexity of the organs. Using cell cultures in microfluidic structures creates a cellular micro-architecture and cellular microenvironment, which influences cell responses. Organs-on-a-chip are devices that allow studying several *in vitro* biological process (physiologic and pathologic) that were not possible to observe using conventional cell cultures systems or animals models^12, 13^. From this technology, organs-on-a-chip produced various models of cell culture in 3D, mimicking organs like lungs^14^, liver^15^, kidney^16^, cornea^17^, neural networks^18^, and models for pathology studies, like breast cancer^19^. These models of organ-on-a-chip enable tissue-tissue interface like epithelial/mesenchymal or parenchymal cells/endothelium, thus allowing tissue interaction through chemical communication, nutrients, hormones, metabolites, cytokines, physical signals and physiological fluidics forces, representing with more accuracy what occurs in the living organ^1^.

Duffy and colleagues^20^ described for the first time a procedure to fabricate closed microfluidics systems using a polymer, which became the most popular fabrication of microfluidic devices, poly(dimethyl siloxane) (PDMS). This technique allowed the production of microdevices in less than 24 h, which represented a significant reduction in the time of fabrication in comparing to glass microchip production.

In the search for alternative materials and easier methods of fabrication has been vast and Thompson and colleagues^21^ reviewed in great detail the polyester-toner microfabrication as viable process to produce microfluidic devices that were simple to produce and of low cost. The PT method uses a layer of toner deposited on a polyester film defining the microfluidic channels; in addition, the toner serves as an adhesive for sealing the device through a step of hot lamination. Thus, the microchips of polyester toner (PT) represent a very promising platform for chemical and biochemical analyses^22–24^.

Using this approach, several types of PT microchips were created for DNA analyse^25, 26^, enzymatic analyses for protein, glucose and cholesterol colorimetric detection in serum samples^27^, and pharmaceutical analyses^28^. Herein we report an alternative model for cell culture under perfusion that mimics a blood vessel, which is intended to study inflammatory response and toxicity thus providing better results than animal models (poor human prediction) or conventional cell cultures (non-3D cell architecture).

## Material and Methods

### Cell line and media

Human Umbilical Vein Endothelial Cells (HUVEC) (CRL-2873™; *ATCC* ^*®*^, *US*) were grown in RPMI-1640 (*Sigma-Aldrich, US*) supplemented with 10% fetal bovine serum (FBS) and 1% mix of antibiotics/antifungals (*Sigma-Aldrich, US*) in polystyrene cell culture flasks (*Corning, US*). The cultures were kept at 37 °C in a humidified atmosphere at 5% CO_2_ and removed from the flask with trypsin (Trypsin-EDTA solution; *Sigma-Aldrich, US*).

### Characterization of the cytotoxicity of the microchip coumpounds

The cytotoxicity of the microchip components was evaluated by the indirect contact assay^29^, where the cells were cultured with extracts from the materials that make up the microchip, *i.e*., cell medium that had been in contact with each material individually. For that, 3 cm^2^ of each material used in the microchip (polyester foil, polyester-toner, and epoxy glue) was soaked in 1 mL of complete medium and incubated for 24 h. before starting the cytotoxicity assay. HUVEC were seeded in a 96 well plate (5 × 10^5^ cells mL^−1^) and cultured for 24 h at 37 °C and 5% of CO_2_. After that, the media was replaced by extracts. Fresh medium was the negative control (C−) and the medium containing 1% sodium dodecyl sulfate (SDS; *Sigma-Aldrich, US*) was the positive control (C+). Subsequently to this, cell viability was tested with methyl thiazolyl tetrazolium (MTT) assay kit (*Sigma-Aldrich, US*). For this purpose, cells were incubated with 1 mg mL^−1^ of MTT for 3 h. The formazan formed was dissolved in 2-propanol and the absorbance was measured using a 540/620 nm filter. The absorbance of untreated cells represented 100% of cell viability^30^.

### Nitric oxide (no) production

To evaluate a possible cellular inflammatory reaction due to contact with the different materials from the microchip the total production of NO was determined in culture supernatant of HUVEC at 5 × 10^5^ cells mL^−1^ incubated for 24 h, 37 °C, 5% CO_2_ with the same concentrations described in cell viability section. As a positive control for the production of NO, recombinant human TNF-α (R&D Systems) (10 ng mL^−1^) was added. On a 50-µL aliquot of cell culture supernatant we added 50 µL of Griess solution (0.1% N-1-naphthylethylenediamine, 1% sulfanilamide in solution of orthophosphoric acid 2.5%). After 10 min of incubation at room temperature protected from light, the absorbance was measured in a spectrophotometer (*Multiskan Ascent, Labsystems*) using 540 nm filter. The concentration of NO released in the supernatant of the cell culture was calculated from a standard curve previously obtained with known molar concentrations of NaNO_2_ in medium supplemented RPMI, and the values were expressed as μmol of nitrite^31^.

### Polyester-toner microchip fabrication

**Figure 1:**
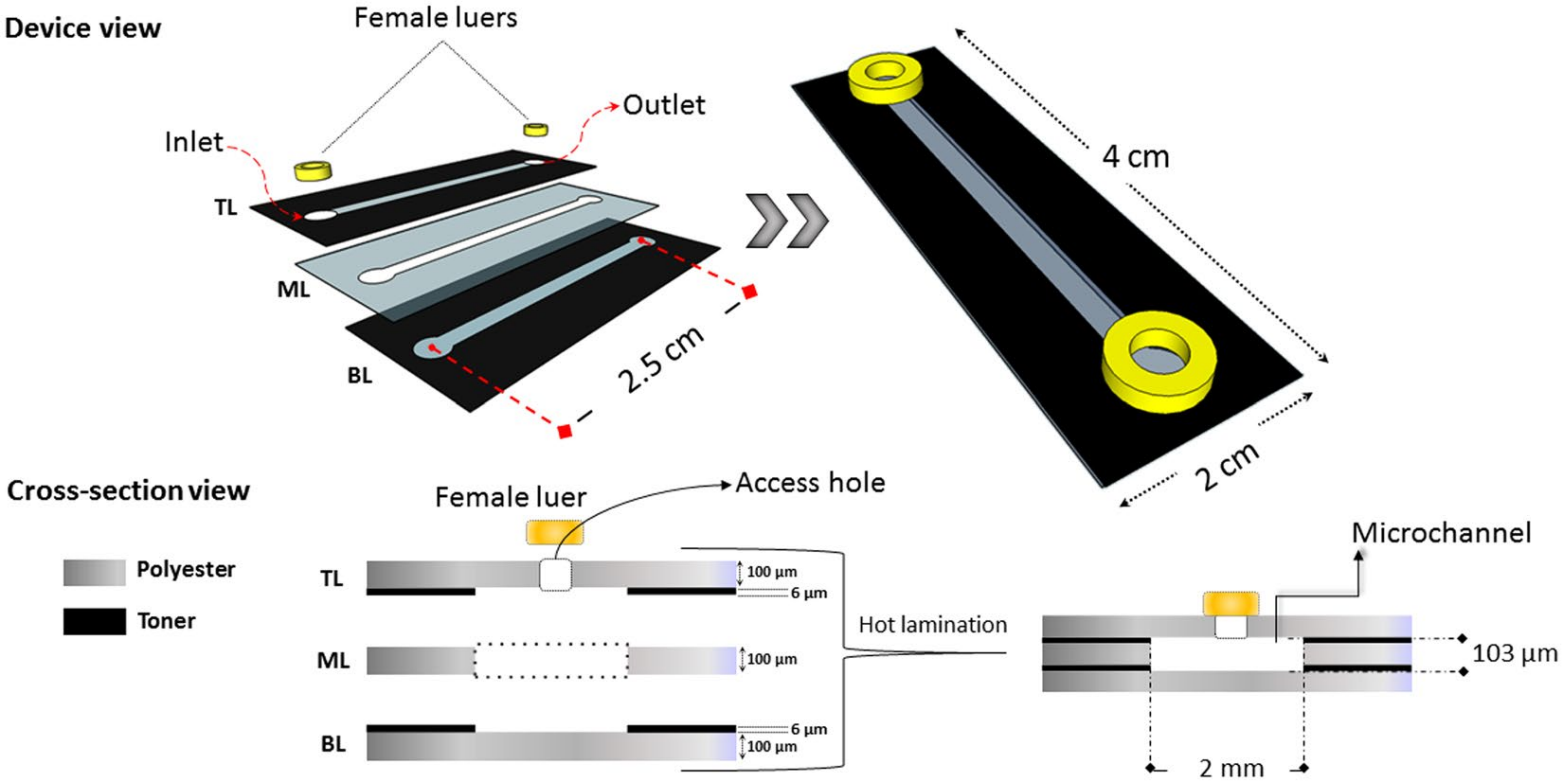
Design, dimensions, and microchip assembly structure. The inner side of the bottom layer (BL) was produced printing the polyester film with one layer of toner creating a channel of 2.0 cm long and 0.2 cm wide and two circles of 0.3 cm of diameter at each end. The top layer (TL) has the same layout printed in the inner layer, but with the circles punched, BL and TL were treated with oxygen plasma and the three layers were laminated together. The channel height was defined by image printing with toner layer (~6 μm) applied over two polyester films that formed the TL, BL of the microchip, and an additional intermediary layer of polyester (middle layer - ML ~100 μm, which was cut through with a lases cutter). Due to the hot lamination step the microchannels height was approximately 103 μm as a result of the toner deformation. Female luers adapters were glued with epoxy resin on the channel end holes.

We chose to make a microchip with a simple design in this study using a straight channel to mimic a segment of a large blood vessel. The PT microchip was fabricated accordingly to the print, cut and laminate (PCL) approach described by Thompson and colleagues^21^. Briefly, the microchip was designed in CorelDRAW^®^15 software (*Corel Corp., Canada*), and printed on polyester films using a LaserJet printer (*1200 series; Hewlett Packard, US*) operating at 600 dots per inch (dpi). The chip was treated with oxygen RF plasma at 50 W and 70 mtorr for 2 min (*Plasma ETCH – Carson City – NV USA*. Since the channel depth is determined by the thickness of the printed toner layer (~6 μm), an additional intermediary foil layer (~100 μm) was used to provide sufficient depth to the channel. For this, the same microchannel layout drove the CO_2_ laser cutter over the polyester film. After that, at to the top of the microchip, inlets of 0.3 cm of diameter were punched to provide access to the microchannel. Subsequently, the three films layers were aligned and laminated together by using an AC 00–1230 laminator (*Gazela, Brazil*) operating at 120 °C and 40 cm min^−1^. Finally, female luers were glued with epoxy resin on the channel access holes. Figure 1 shows the microchip assembly procedure, dimensions, and structure of the chip.

### Microchannel surface analysis after oxygen plasma modification

The surface hydrophilicity imposed by oxygen plasma treatment was evaluated by sessile drop contact angle measurements with three different liquids, water, formamide, and ethylene glycol. Three measurements on different points were performed on each sample and the estimated error was found to be ±3°. They were carried at room temperature in a CAM 200 goniometer (KSV, Finland) and the images were analyzed using CAM 2008 software (KSV, Finland). Surface free-energy (γ_s_) values as well as the dispersive (γ_s_^d^) and polar (γ_s_^p^) components were estimated according to the Owens-Wendt model^32^.

### Topographical study

A Nanosurf easy Scan 2 AFM System (*Nanosurf Switzerland*) operating in non-contact mode atomic force microscopy (AFM) obtained images for surface rugosity with a sample size of 50 μm^2^. The silicon AFM probes operated under a constant resonance frequency of 190 kHz.

### Biological modification/preparation of the channel surface

To promote cellular adherence and the endothelialization of the device, the microchip was treated with 50 µL solution of fibronectin 10 µg mL^−1^ in PBS (*Fibronectin from bovine plasma – Sigma-Aldrich, US*), and incubated for 72 h, 4 °C, and dried under 37 °C for 120 min.

### Cell seeding and cultivation in microchip

**Figure 2:**
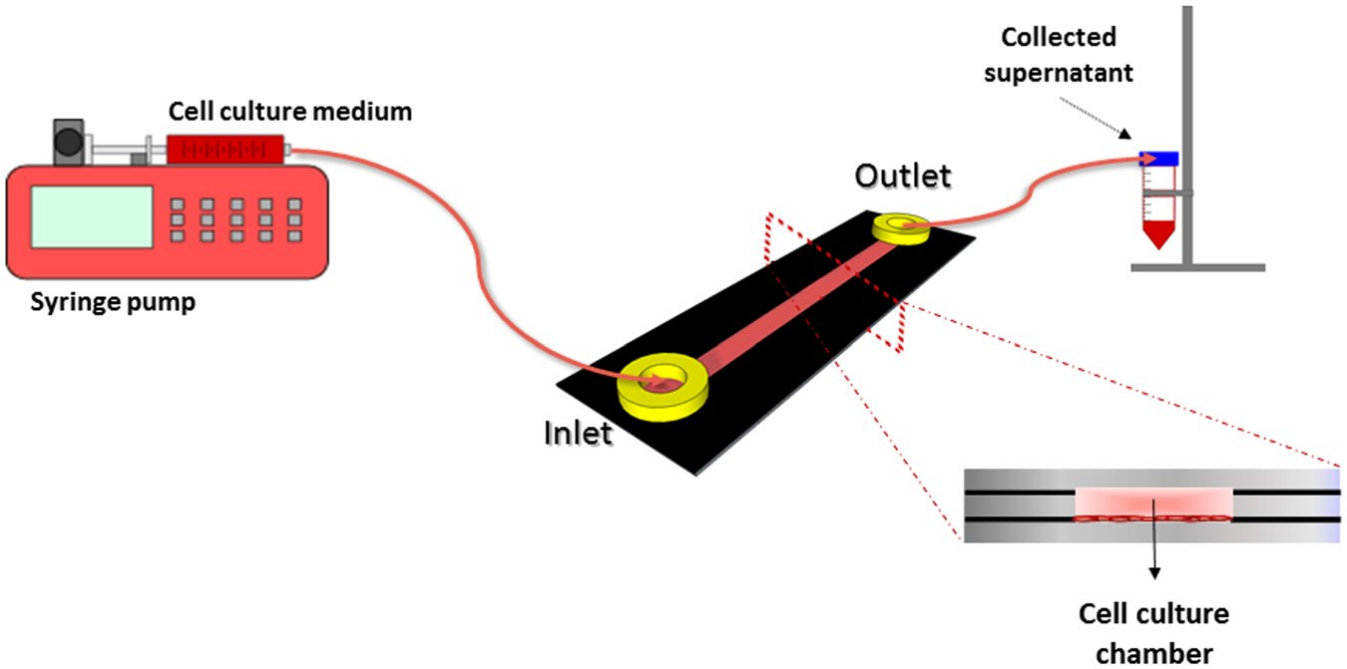
Schematic depiction of the microchip dynamic system. After cell adhesion to the microchannel for 4 h under static conditions in incubator (a crucial point in the ability of the cells to resist to the subsequent shear stress), the microchip was connected to a syringe pump. The microchip was perfused at a continuous unidirectional flow rate of 2 μL min^−1^ with culture medium RPMI supplemented with 10% FBS and 1% mix of antibiotics/antifungals and connected with silicone tubes to the perfusion system. The syringe was attached to the PT microchip through a female luer adapter and the supernatant was collected on the opposite access. The outlet access was attached to a sterile Falcon^®^ tube, which was sealed with plastic film (Parafilm^®^ “M” – Laboratory Film). The system, composed by the microchip and a pump, stayed in the incubator at 37 °C, in a humidified atmosphere at 5% CO_2_ during the course of the experiment.

The microchannel was filled with 150 µL of the cell suspension at a concentration of 5 × 10^6^ cells mL^−1^. To help the cell suspension flowing through the channel, the microchip was inclined slightly. Caps were put on the luer adapters and the microchip was placed into an incubator (37 °C, 5% CO_2_) for 4 h, to allow the adherence and spreading of the cells along the microchannel under static conditions. After this period, a semi-confluent and out stretched cell monolayer was established. Subsequently, a syringe pump perfused the microchip at a continuous unidirectional flow rate of 2 μL min^−1^ with culture medium RPMI supplemented with 10% FBS and 1% mix of antibiotics and antifungals. This system, composed of the microchip and a syringe pump (Fig. 2), was kept in an incubator at 37 °C, in a humidified atmosphere (by means of a water tray inside the incubator) at 5% CO_2_ during the experiment. Besides, a static control microchip was incubated at the same conditions afore mentioned. The cultured cells were imaged every 24 h using an inverted microscope (CKX41; Olympus, Japan) equipped with a CCD camera.

### Determination of vegf-a by immunoassay test

An aliquot of supernatants from HUVEC cell perfusion was collected every 24 h (end time: 72 h), centrifuged at 300 × g for 10 min and stored at −20 °C until the determination of VEGF-A. For comparison, the same cell concentration used in the microchips grew on 24-well polystyrene plates. An ELISA assay (*BD Biosciences, US*) measured VEGF-A using the protocol provided by the manufacturer.

### Viscosity and shear stress calculation

The viscosity of culture medium containing 10% FBS was determined in automatic micro-viscometer (AMVn - Anton Paar). Shear stress (*τ*) inside the microchannel during the culture was estimated according to the equation 1:

$$\tau =\frac{2\mu Q}{w{h}^{2}}(\frac{m+1}{m})(n+1)$$

where *µ* is the viscosity of the medium, *Q* is the volume flow rate, *h* is the height of the microchannel, *w* is the width of the microchannel, and *m* and *n* are empirical constants, with *m* = 1.7 + 0.5(*h/w*)^−1.4^ and *n* = 2 for aspect ratios *h/w* < 1/3^33^.

### Statistical analysis

All results were expressed as the mean value ± one standard deviation of the mean for three independent assays (n = 3). To assess the level of significance, we performed a one-way ANOVA followed by a Tukey test for multiple comparisons. Besides, comparisons against the control experiment used one-way ANOVA with two-sided Dunnett’s test. Value of *p* < 0.05 was considered to be statistically significant in all cases.

## Results and Discussion

**Figure 3:**
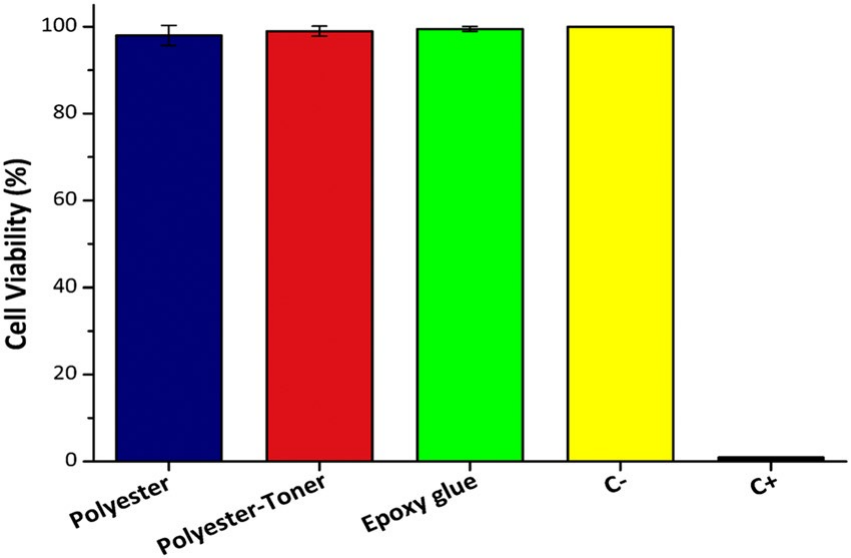
Cell viability using microchip materials extracts as the medium being supplied to the cells (indirect method) and allowed them to grow. C− (negative control): fresh culture medium. C+ (positive control): SDS 1%. Cell count per well: 5 × 10^5^. Culture conditions: 24 h, 37 °C, and 5% of CO_2_. Results are expressed as average viability and the error bars represent one standard deviation of viable cells (both in % values). Statistical analysis was performed using one-way ANOVA with two-sided Dunnett’s test (*p < 0.05 with respect to negative control).

Prior to the microchip assembly, we investigated the cytotoxicity of the microchip materials (polyester foil, polyester-toner, and epoxy glue) by isolating each one of them and testing for cell viability. The MTT test showed cell viability in all wells where HUVEC cells were challenged against the microchip materials. No significant cell death was observed when we compared cell viability to the negative control (Fig. 3).

The Griess method revealed that NO was not detected in the supernatant of HUVEC cells cultured for 24 h with each component of the microchip (polyester, polyester-toner, and epoxy resin). However, there was a statistically significant increase in NO production in the positive control (TNF-α at 80 µmol mL^−1^) thus validating this test (data not shown). The materials used in the microchip construction demonstrated to be neither toxic nor caused significant cell death, thus the microfabrication of the chip was initiated without worries about toxicity of any material used in the microfluidic system.

Several main properties of ECs that are affected by flow-induced mechanical forces, including adhesion strength, migration, adaptive response to shear stress, and permeability^33^. These forces are imposed directly on the apical surfaces of the ECs, and are transmitted to the cell cytoskeleton via transmembrane mechanosensors at the surface^33, 34^. The cytoskeleton is also linked to integrins found on the basal surface of ECs^35^. The cytoskeleton therefore forms a bridge between mechanosensors on the luminal side and the integrins on the abluminal side, which have been described as both mechanotransducers^35^ and mechanosensors^36^. All endothelial cells (ECs) *in vivo* are exposed to blood flow-induced shear stress^33^—a mechanical force, which is tangential to blood vessel wall—produced by friction with the viscous blood flow. The shear stress acts as a structure modulator of the blood vessels and their functions^37^. Shear stress has been shown to mediate endothelial morphological adaptations^38–40^, endothelial permeability^41^, vasoregulation^42^, arterial remodeling^43, 44^, and pathophysiological processes leading to atherosclerosis and other cardiovascular diseases^45^. Due to its importance, shear stress effects on endothelial function have been studied extensively in the last forty years, both *in vivo* and *in vitro* ^33^.

Viscosity (*µ*) of RPMI culture with SFB 10% was 7.7 × 10^−3^ g cm^−1^ s^−1^. Volume flow rate of 2 µL min^−1^, *h* = 103 µm and *w* = 200 µm, shear stress was 4.48 dyn cm^−2^. The majority of studies use steady shear stress in the range of 10 to 40 dyn cm^2^ to mimic physiological conditions^33, 46^, consequently, in our system the shear stress over the cells can be considered low. Due to the low shear stress developed in our microchip, the HUVECs were elongated and they showed polygonal conformation, but they did not show a defined alignment to the direction of the flow. Similar behavior was observed by Song and co-workers^47^ who measured EC morphological response and obtained data on elongation and orientation consistent with the literature, *i.e*., low shear of 1 dyn cm^2^ did not align cells while high shear of 9 dyn cm^2^ aligned cells parallel to the flow. Nevertheless, due to the variety of experimental conditions, data cannot be easily compared to the large body of literature on steady shear EC responses.

**Figure 4:**
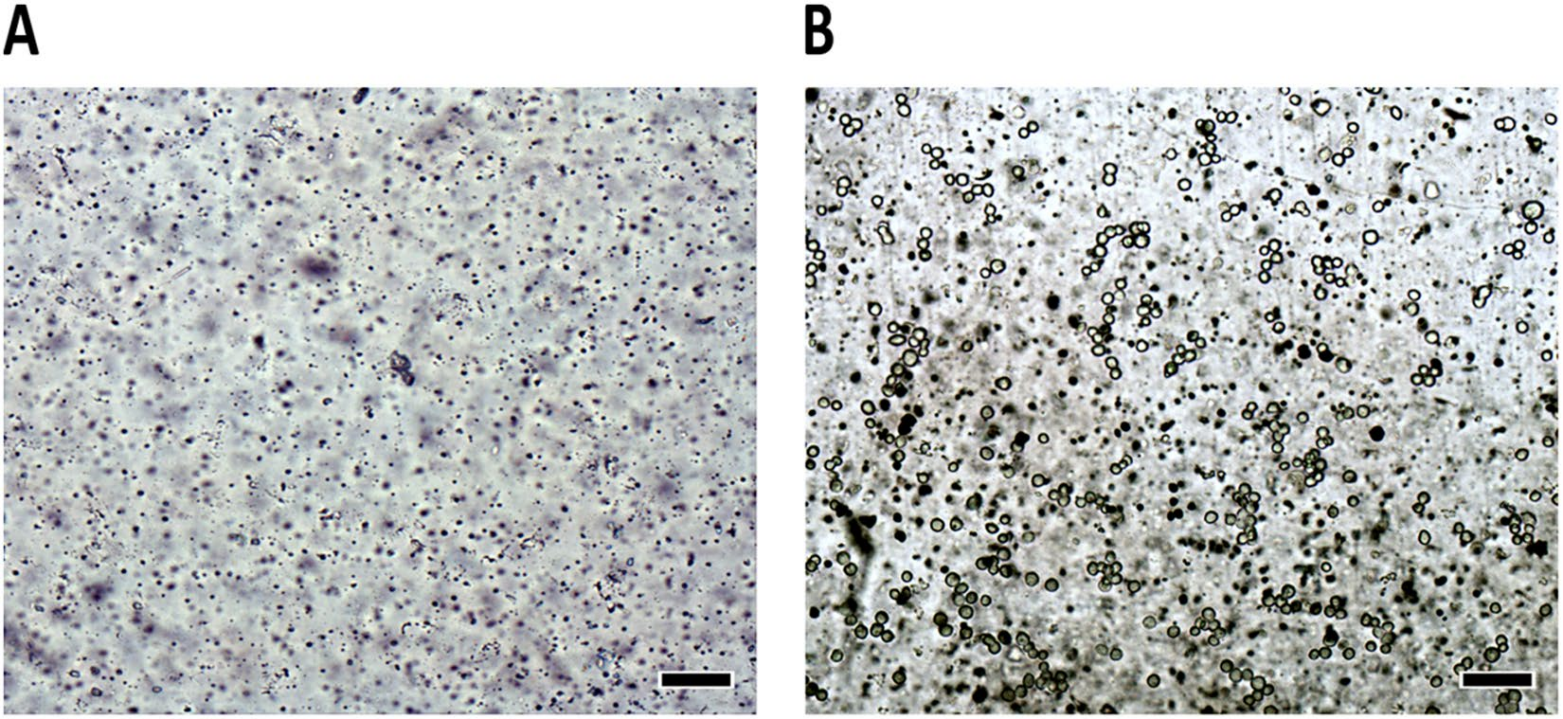
Micrographies of the microchip before and after cell injections. **(A**) Untreated PT microchip channel before cell introduction (the image illustrates the roughness of the polyester surface and the dark spots are artifacts from scattered toner particles). (**B**) Untreated PT chip with cells (circles with a clear center) after introduction of 5 × 10^6^ HUVEC cells mL^−1^ into the microchip (time zero). Magnification: 100× ; scale bar: 100 µm.

Each cell suspension (5 × 10^6^ cells mL^−1^) injected into the microchip (dynamic and static) were observed, evaluated, and photographed every 24 h. Figure 4 shows the micrographs of the channels before (Fig. 4A) and after (Fig. 4B) injection of the cell inside the microchip (establishing time zero for the growth curve). We can observe that the cells were spherical and refringent at the start of the experiment.

**Figure 5:**
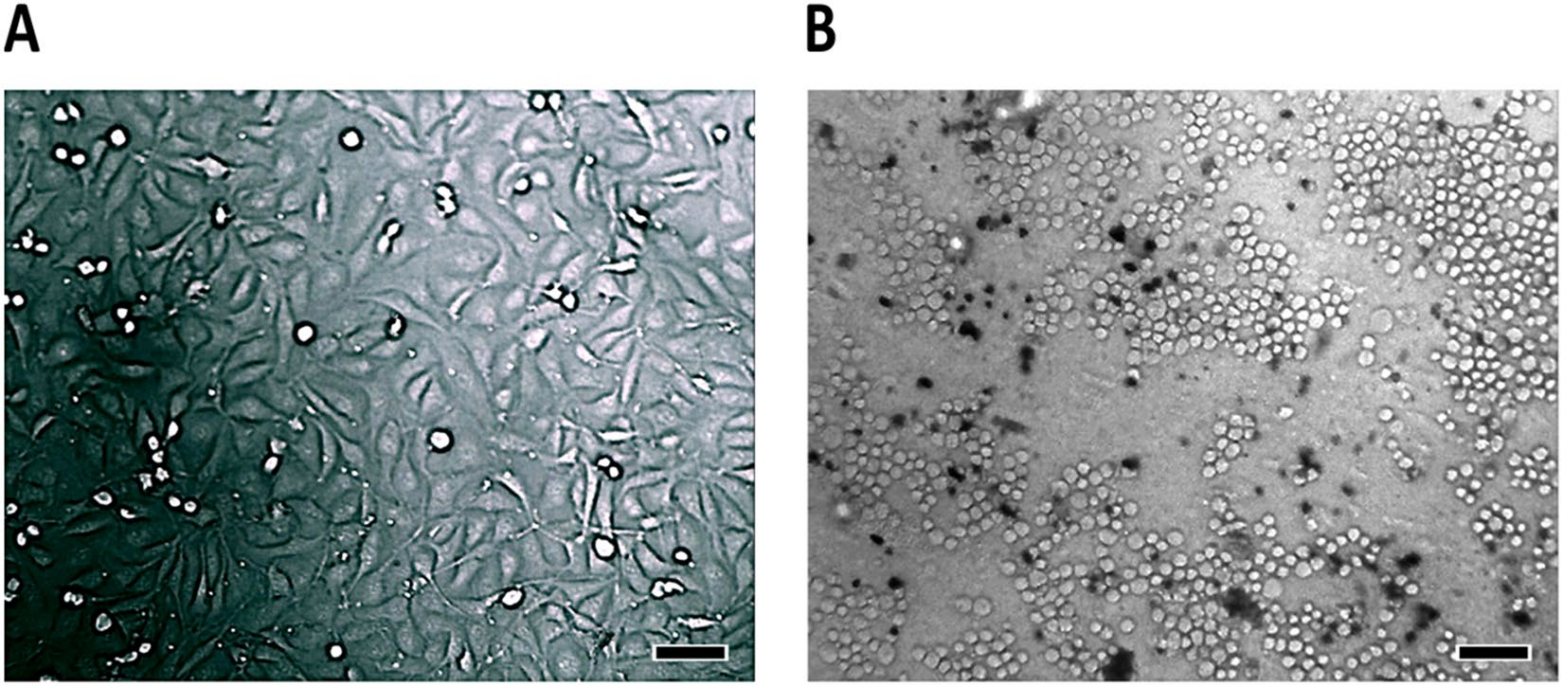
Morphological evaluation of HUVEC cells after 24 h growth in different environments: **(A**) polystyrene bottle and **(B**) untreated PT microchip. Magnification 100× ; Scale bar: 100 µm.

Morphological differences of HUVEC cells grown into the untreated microchip (not treated with oxygen plasma and/or fibronectin) and perfused for 24 h with RPMI medium are easily visible when compared to the cells that were subjected to conditions of cultivation in polystyrene bottle or plate. Cells cultured in polystyrene flask present a confluent elongated shape (Fig. 5A), while those that were grown in the untreated PT microchip remained spherical and non-confluence (Fig. 5B).

Polyester does not promote cell adhesion, as we can observe comparing cell morphology and lack of cell confluence between cells grown in the microchip and those grown in a flask (or in a plate - data not shown). To increase the interaction between the cells and the polymer surface, it is necessary to establish that the protein adsorption and subsequent cell adhesion to the substrate is determined by the substrate properties that involve ionic interactions, hydrogen bonds, and electrostatic forces. Even though the substrate may not necessarily have the same characteristics of the extracellular matrix at which cell adhesion takes place, the physicochemical similarity is desirable when the objective is to promote cell differentiation^48, 49^. It is only after cells begin to adhere that the process of spreading, division, and production of new extracellular matrix begins^50, 51^. Cell spreading is a complex process that involves changes in cell morphology as a result of alterations in its cytoskeleton, thereby, improving interactions with the substrate^51^. Several studies have shown that surface properties of a material can be modified to promote better interactions with cells^52–54^.

In the microchip that was treated with oxygen plasma and perfused with culture medium, some of the HUVEC cells presented morphological aspects that were similar to those that were grown in bottles and polystyrene plates such as: cells in the stretching process or that are already elongated (data not shown). This observation is evidence that polyester treatment with oxygen plasma promotes cell adhesion to the surface, thus, promoting a more elongated shape. In some treated and perfused microchips however, not all cells acquired an elongated shape nor presented 100% confluence inside the microchip, even after 48 h of experiment, what required further action.

**Figure 6:**
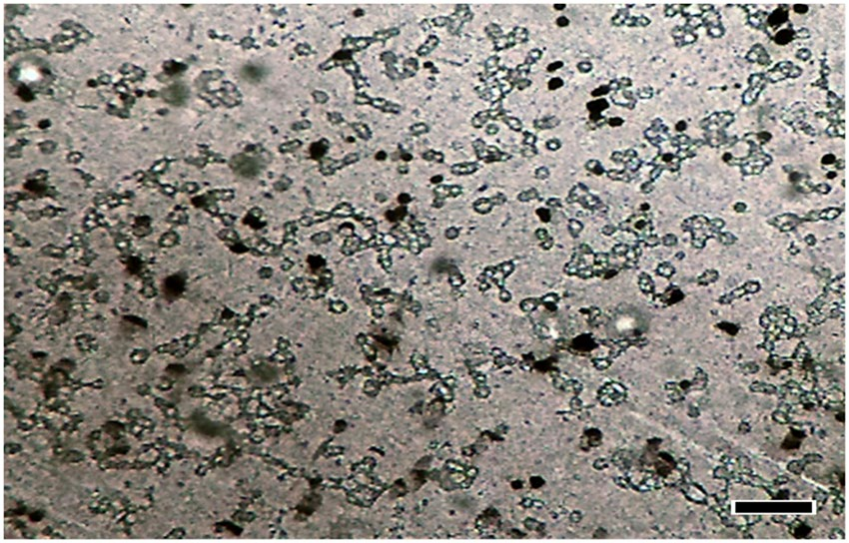
Cells grown on a static PT Microchip (treated with oxygen plasma). Microchip incubated for 48 h at 37 °C and 5% CO_2_ without perfusion. Initial cell concentration: 5 × 10^6^ cells mL^−1^. Magnification 100× ; Scale bar: 100 µm.

When we analyzed the static microchip (Fig. 6) that remained in the incubator throughout the test, we did not observe any morphological transformation on the cells going from spherical to elongate. Continuous flow of the medium influenced positively cell development, thus, demonstrating the importance of microfluidics on maintaining cell survival within the microchip. Cells on the static microchip were slightly refractive, smaller and, with loss of initial morphology, suggesting they were in the process of dying.

**Figure 7:**
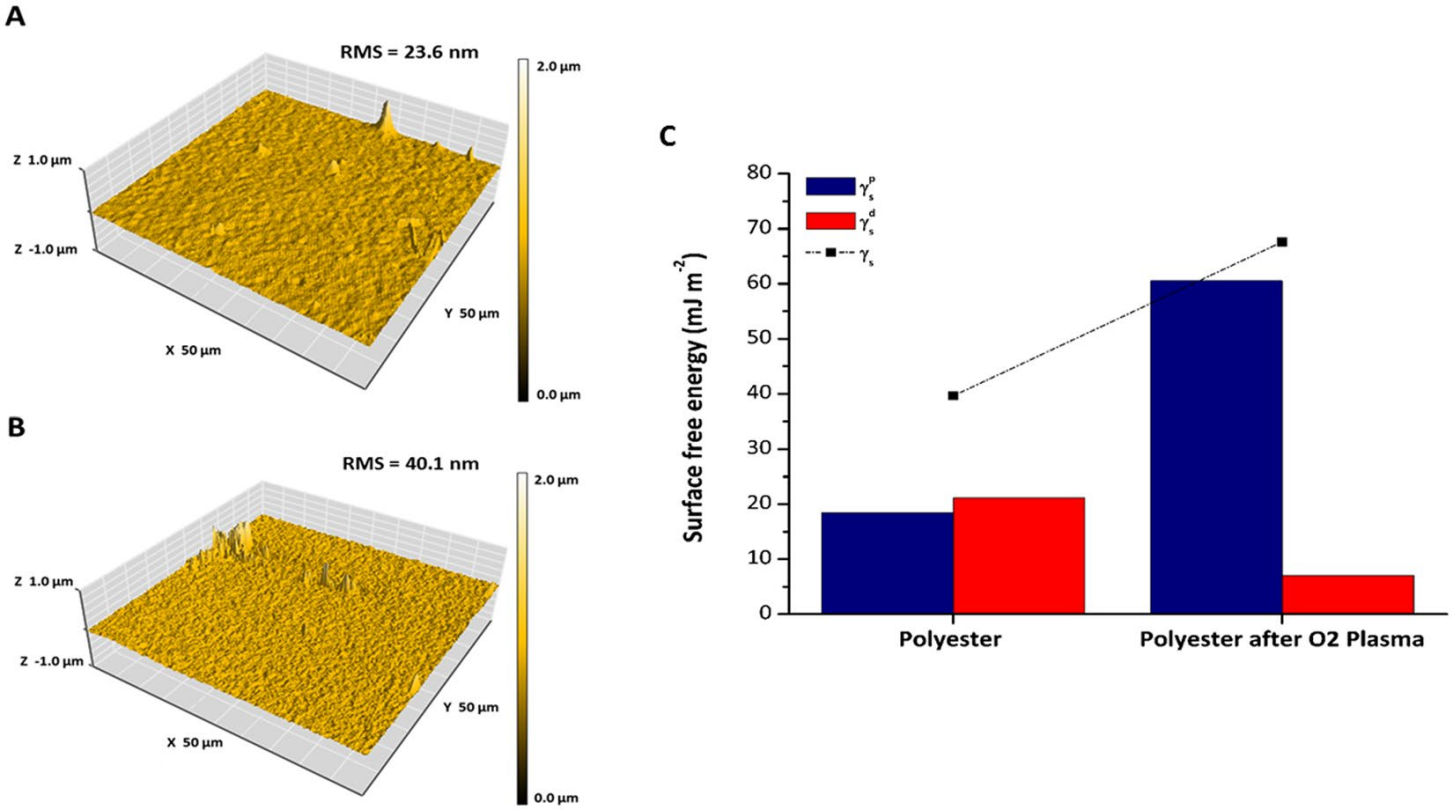
Characterization of the surface roughness, energy, and cell viability upon treatment with oxygen plasma. Atomic force micrographs of the polyester film before (**A**) and after (**B**) oxygen plasma treatment, showing changes in the rugosity of the surface. RMS: root mean square of average rugosity (nm). (**C**) Evaluation of the change in surface free-energy of the polyester surface before and after treatment with oxygen plasma. The native polyester film presents a polar component of 18.4 (mJ m^−2^) this value changed to 60.9 (mJ m^−2^) when the polyester film was treated. γ_s_: Surface free-energy. γ_s_^d^: dispersive component of surface free-energy. γ_s_^p^: polar component of surface free-energy. The estimated error for the contact angle measurements was ±3° for n = 3.

To improve cell adhesion, the polyester used in our microchip, after printed with a toner layer, was treated with oxygen plasma because it causes major changes in the polarity of the surface of the film as demonstrated in Fig. 7A and B. In addition, plasma treatment sterilized the surface of the microchip destroying microorganisms, removing bacteria, fungi, and viruses^55^. Figure 7 shows the rugosity of the polyester without (A) and with (B) oxygen plasma treatment. The average rugosity difference observed between them was 16.5 nm, thus evidencing an increase on the rugosity in the treated polyester favoring cell adhesion. Our results are consistent with studies showing that rougher surfaces promote cell adhesion and proliferation^56, 57^.

Energy and rugosity of a surface are important parameters that affect cell growth, adhesion, and differentiation^58^. After the oxygen plasma treatment, we observed changes on the polyester film that improved cell adhesion and growth. The surface modification of the polyester significantly increased the surface energy or wettability, as demonstrated in Fig. 7C. The O_2_ plasma treatment led the polyester films to a further increase in the hydrophilicity over the native film confirmed by the increase in total surface free energy. While the native polyester film presented a polar component of 18.4 mJ m^−2^, this value changed to 60.9 mJ m^−2^ when the polyester film was treated. We assume, therefore, that the plasma treatment induces chemical modifications on the surface of polyester films, producing hydrophilic/polar groups.

When the microchip was treated only with oxygen plasma, the cells kept adhered to surface for 2 or 3 hours, but then released, in both static and dynamic systems. When the microchip was not treated with oxygen plasma but only fibronectin, the cells also kept adhered for 2 hours maximum. The best condition for cell adhesion is the combination of oxygen plasma with fibronectin. Without physical or biological modification on the surface the cells do not adhere in polyester.

**Figure 8:**
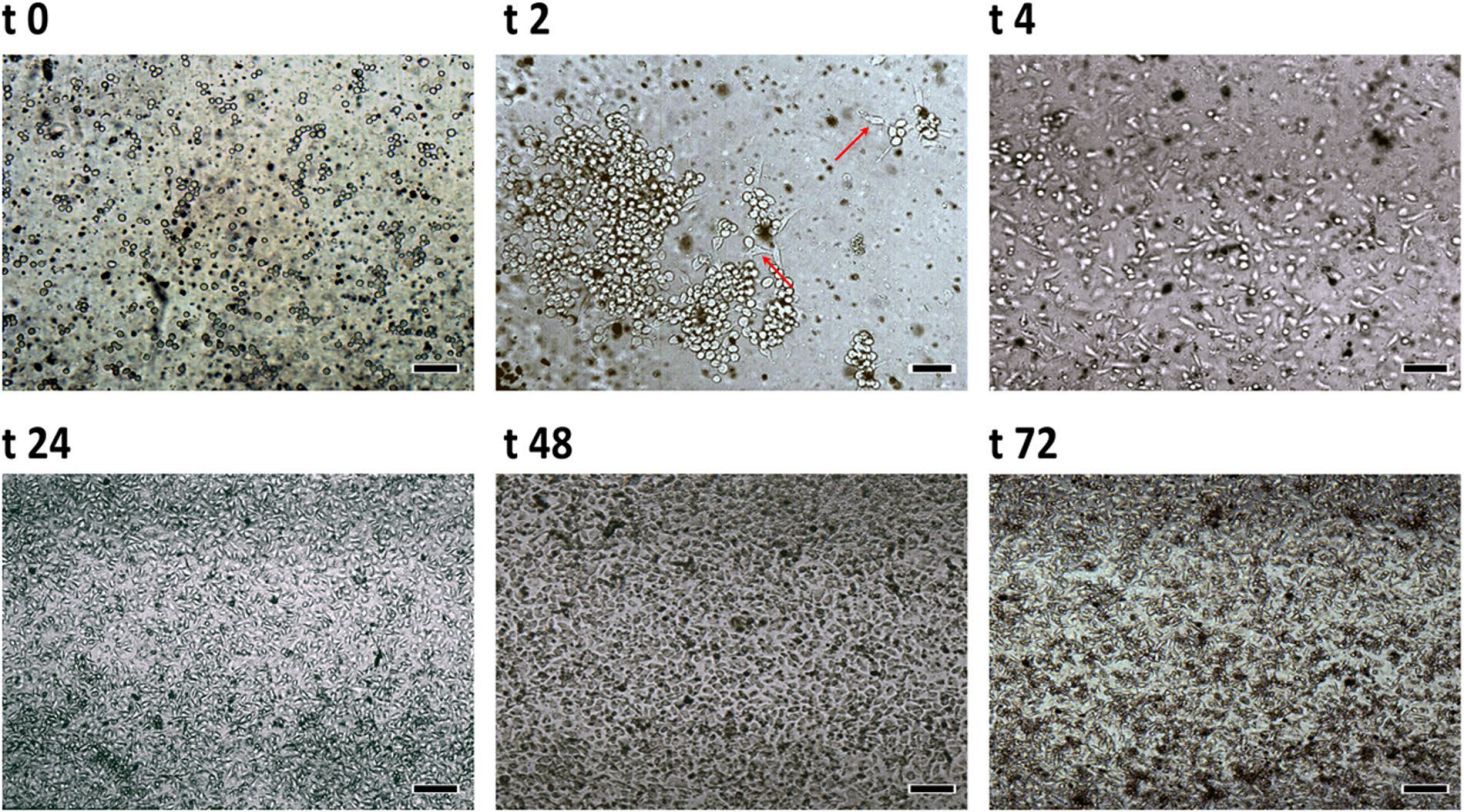
Cell development inside the PT microchip treated with oxygen plasma and fibronectin before (t0 – t4) and after perfusion in function of time (t24–t72). t0: time zero – without perfusion; t2: 2 h after cell introduction in microchip but before perfusion (arrows indicate elongation and branching in the beginning of HUVEC spreading); t4: 4 h after cell injection in microchip but before perfusion. t24, t48 and t72: 24, 48 and 72 h, respectively of perfusion of RPMI medium by the syringe pump at a flow rate of 2 µL min^−1^. Initial cell count: 5 × 10^6^ cells mL^−1^. Magnification 100×; scale bar: 100 µm.

When the microchip surface was treated with oxygen plasma and fibronectin, we observed an increase of cell adherence and cell confluence (Fig. 8), which is in agreement with previous findings from Strom and colleagues^59^ and Pompe and collaborators^60^. Cells are inherently sensitive to physical, chemical, and biochemical stimuli present in the microenvironment^61–63^. *In vivo*, cells are in intimate contact with the extracellular matrix (ECM), which is formed from binding complex proteins, glycoproteins, and proteoglycans.

The ECM not only provides structural support but also contains a variety of motifs for cell signaling and growth factors that guides cell adhesion and cell behavior. Cellular microenvironment provides an environmental stimulus that determines specific cell behavior, including selective recruitment, proliferation, differentiation, and production of numerous proteins necessary for the hierarchical organization of the tissue^53, 64^.

The ECM is composed of several macromolecules (fibril proteins such as collagen and glycoproteins) at different concentrations that interact with proteins on the cell surface, and soluble macromolecules such as growth factors^65^. In addition, organization, density, spatial geometry and biochemical characteristics of these, ECM components determine mechanical strength, cellular response, cell adhesion, and hierarchical organization of the tissue^53^. Some ECM-derived proteins, such as collagen, fibronectin, laminin, and vitronectin are described as facilitating agents for adhesion of various cell types because they possess intrinsic biological recognition sites via integrin receptors^53, 66^.

The process of endothelialization consists on mimicking the extracellular matrix thus improving cell adhesion and therefore, providing an ideal condition for cells to assume their native morphology (which is elongated), promoting the endothelialization of the device, which was treated with a solution of fibronectin concentration 10 µgmL^−1^. Figure 8 shows the development of the cells inside the microchip treated with oxygen plasma and fibronectin after continuous perfusion over time.

In this experiment we demonstrated the great importance of oxygen plasma treatment, fibronectin, and microfluidics to promote the spread, the adhesion, and the survival of HUVEC cells in PT microchips, making the combination of these components mandatory for cell studies and further mimicking organs.

Figure 8 shows that 2 h after cell injection into the microchip some cells throw cytoplasmatic elongations towards the others (see t2 and t4) and several cells changed from a acinar shape (t0) to a more elongated one, similar to the shape found in cells grown in plates/bottles of polystyrene. However, when the microchip was maintained at 37 °C without perfusion, cells became elongated in the first 4 hours but returned to a more acinar shape after 24 h; at 48 h cell shape was similar to that of cells in Fig. 8 indicating a reversal of the process of differentiation (data not shown). These results indicate that polyester treatment with oxygen plasma and fibronectin promote cell adhesion and cell survival but still depends upon a constant flow of culture medium. We also observed that after 24 h of continuous perfusion of the medium, cells showed 100% confluence and the microchip channel was completely filled with cells, and after 48 h, a very high number of cells filled the microchip. Previous tests carried out in our laboratory demonstrated that microchips not treated with fibronectin did not allow cell attachment (data not shown). Also from Fig. 8 we observed that cells became elongated in shape, without a preferable direction, due the low shear stress developed in the microchannel. Additionally, we also observed that when cells are injected with culture medium but without supplementation with magnesium and manganese cell adhesion is not as efficient as when these elements are present, thus demonstrating the importance of these elements for cell adhesion and proliferation.

The different cultures in the microchip were viable for 5 consecutive days, after this period the cells started to lift off from the surface. In comparison with traditional cultures the chip is advantageous because the culture medium is always renewed. In traditional culture medium if the cells do not have the culture medium renewed constantly, at least every 2 days, the pH changes and compromises the viability and the cellular function. Therefore, the dynamics of the fluids on the device is advantageous to the cells. After 5 days, occurs the detachment of the cells due to the lack of space for the new cells to adhere and this ends up creating a cell mass that is created on top of the first cell layer.

Integrins are a family of cell surface molecules that mediate adhesive interactions with the ECM, activating important intracellular pathways for many biological processes including adhesion and differentiation^67, 68^. Some functions of the integrins are dependent on the interaction with divalent cations such as calcium, magnesium, and manganese through site adhesion dependents upon a metal ion (*Metal Ion Dependent Adhesion Site* – MIDAS) or a MIDAS-like motive^69–71^. The magnesium is considered the most important element for cell adhesion by increasing the affinity between integrins and other ligands of the ECM^71^.

**Figure 9:**
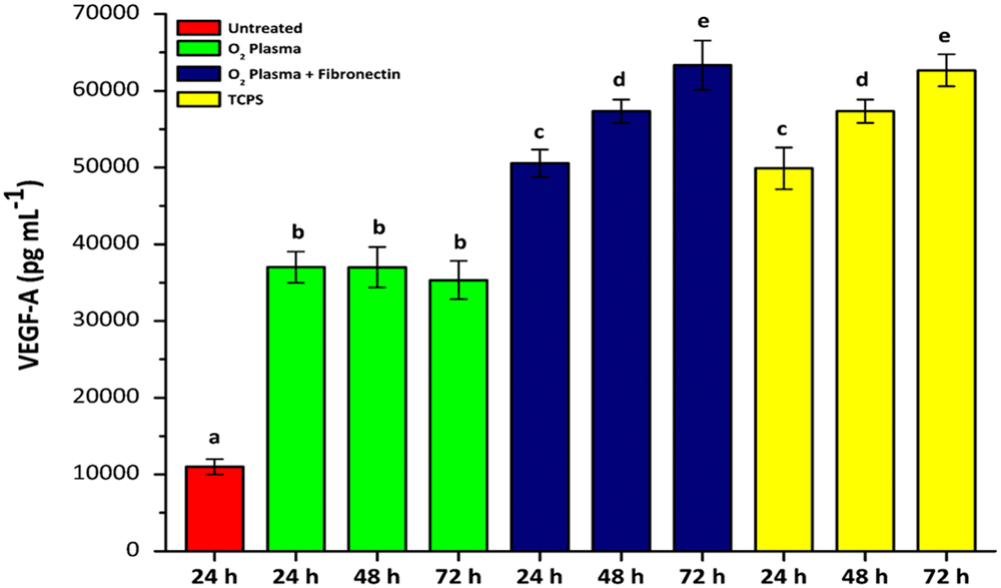
Levels of VEGF-A production assay for 24, 48 and 72 h from cells grown in microchips subjected to different treatments and the comparison with the reference culture growth in polystyrene plates. Initial cell count in the microchip and plate well: 5 × 10^6^ cells mL^−1^. TCPS: Tissue Culture Polystyrene Bottle. Results presented as mean ± standard deviation (n = 3). Statistically significant differences (*p* < 0.05) are represented by different letters above the bars.

VEGF-A enhances cell adhesion and also activates integrins^72^. The VEGF-A was measured in the supernatant of the RPMI 10% FBS medium that perfused the microchip, treated with oxygen plasma and fibronectin, to check its influence on cell adhesion. VEGF-A production was also measured in the supernatant of the static culture that was grown in polystyrene plates. The results are shown in Fig. 9.

Results for the production of VEGF-A in the supernatant showed that its concentration is significantly lower (*p* < 0.05) in untreated microchip than microchips treated with oxygen plasma and/or fibronectin, and also to the levels detected in the polystyrene plate. The results suggest that the adhesion enhancement is closely related to the VEGF-A expression in O_2_ treated polyester films. Besides, a further adhesion was observed when both fibronectin and O_2_ plasma were used together due to the enhanced the adhesion sites for the anchorage of integrin present on the cell membrane. Besides, this fact might correlate with cell adaptation to the microchip substrate favoring the cell adhesion and multiplication. Our results suggest that the chip treatment favors cell growth over native microchips. We also observed that the microchip treatment with oxygen plasma and fibronectin provided a VEGF-A expression similar to that observed in cells cultured in a polystyrene plate, again demonstrating that the treatment was effective for polyester thus promoting better survival and cell growth. Then, it is possible to suggest that the cell adhesion efficiency into the microchip is closely related to the increase of VEGF-A expression to promote neo-vascularization or neo-angiogenesis in this device. VEGF-A has been described as a key regulator of blood vessel growth^73^, thus in an attempt to promote endothelialization of the microchip channel or well plate, HUVEC cells express VEGF-A.

Thus, the developed microchip in this study showed advantages in relation to microchips that were produced with PDMS and glass, because polyester and toner are low cost materials, they can be easily found and fabricated and also reduced time to produce. Besides that, the devices provided the ideal conditions for the cells grow and survive, showing an appropriate environment for the cells in the polyester. Moreover, the materials proved absence of toxicity and good optical transparency to monitoring of the cell growth in microscope. When we evaluated the possibility of producing flow of the medium culture through infusion pump, we were able to create a dynamic system capable of mimicking the observed conditions *in vivo* in blood vessels of small caliber. However, limitations have been also observed in this device, because it was not possible to use higher flow rates, due to the possibility of the microchip can be damaged, and it is necessary a previous treatment of the polyester for existing a better adherence of the cells.

The possibility of cell growth in PT microchips has added a great progress to expand the experience of our group in the development of microfluidic devices with these materials. We and others have previously demonstrated the PT microchips were amenable to DNA analyzes^25, 26^, to enzymatic reactions for protein, glucose and cholesterol detection in serum samples^27^, thus being compatible with biological reactions. We believe that the application of PT in the development of organ-on-a-chip has a great potential to popularize new platforms for cellular studies.

## Conclusions

As new materials for cell biology, polyester-toner showed to be non-toxic to the cells. The microchip treated with oxygen plasma and fibronectin allowed better cellular adhesion and proliferation, while perfusion of the microchip was fundamental for cell survival. Furthermore, hence the microfabrication of the devices proved to be very practical and simple, one would expect that this technology could be replicated in many laboratories, being an alternative to traditional static cell culture. In the future, this platform could also be preferable to using animal models when produced as structured organ-on-chips, with the advantage of being produced at a low cost, easy fabrication, and reduced time in relation to PDMS chips and other more elaborated Microfabrication techniques. The advantages of PT microchips may also include variable structure design, flexibility in microfabrication, and combination of materials, which may facilitate systematic study of “organ-on-a-chip”. The PT biomimetic microchips could be applied to a variety of purposes and this may accelerate the advancement of knowledge in this research area.
